# The Mechanism of Release of P-TEFb and HEXIM1 from the 7SK snRNP by Viral and Cellular Activators Includes a Conformational Change in 7SK

**DOI:** 10.1371/journal.pone.0012335

**Published:** 2010-08-23

**Authors:** Brian J. Krueger, Katayoun Varzavand, Jeffrey J. Cooper, David H. Price

**Affiliations:** 1 Molecular and Cellular Biology Program, University of Iowa, Iowa City, Iowa, United States of America; 2 Biochemistry Department, University of Iowa, Iowa City, Iowa, United States of America; Roswell Park Cancer Institute, United States of America

## Abstract

**Background:**

The positive transcription elongation factor, P-TEFb, is required for the production of mRNAs, however the majority of the factor is present in the 7SK snRNP where it is inactivated by HEXIM1. Expression of HIV-1 Tat leads to release of P-TEFb and HEXIM1 from the 7SK snRNP in vivo, but the release mechanisms are unclear.

**Methodology/Principal Findings:**

We developed an in vitro P-TEFb release assay in which the 7SK snRNP immunoprecipitated from HeLa cell lysates using antibodies to LARP7 was incubated with potential release factors. We found that P-TEFb was directly released from the 7SK snRNP by HIV-1 Tat or the P-TEFb binding region of the cellular activator Brd4. Glycerol gradient sedimentation analysis was used to demonstrate that the same Brd4 protein transfected into HeLa cells caused the release of P-TEFb and HEXIM1 from the 7SK snRNP in vivo. Although HEXIM1 binds tightly to 7SK RNA in vitro, release of P-TEFb from the 7SK snRNP is accompanied by the loss of HEXIM1. Using a chemical modification method, we determined that concomitant with the release of HEXIM1, 7SK underwent a major conformational change that blocks re-association of HEXIM1.

**Conclusions/Significance:**

Given that promoter proximally paused polymerases are present on most human genes, understanding how activators recruit P-TEFb to those genes is critical. Our findings reveal that the two tested activators can extract P-TEFb from the 7SK snRNP. Importantly, we found that after P-TEFb is extracted a dramatic conformational change occurred in 7SK concomitant with the ejection of HEXIM1. Based on our findings, we hypothesize that reincorporation of HEXIM1 into the 7SK snRNP is likely the regulated step of reassembly of the 7SK snRNP containing P-TEFb.

## Introduction

Transcription elongation by RNA polymerase II (RNAPII) is a highly regulated process resulting from the concerted effort of both negative and positive elongation factors. After initiation engaged RNAPII molecules come under the control of negative factors including NELF and DSIF that limit the elongation potential of the polymerases and trap them in promoter proximal positions [1], [2], [3]. These polymerases are poised for release into productive elongation that ultimately generates mRNAs. The positive transcription elongation factor, P-TEFb, is a cyclin dependent kinase that phosphorylates the negative factors and RNAPII leading to the transition into productive elongation [4], [5]. It is clear that P-TEFb plays a key role in the regulation of gene expression because a number of genome wide studies have determined that many genes in Drosophila and most genes in mammals are occupied by poised polymerases [6], [7], [8], [9], [10], [11].

The kinase activity of P-TEFb is required for the generation of mRNAs; however, the majority of P-TEFb is sequestered within the 7SK snRNP where it is inactivated through association with HEXIM1 or HEXIM2 proteins [12], [13], [14], [15]. A mechanism must exist to extract the kinase from this inhibitory complex and direct the function of P-TEFb to specific genes. Release of P-TEFb from the 7SK snRNP could occur through post-translational modification of P-TEFb or components of the 7SK snRNP. One study showed that dephosphorylation of the T-loop of P-TEFb by PP1α and PP2B results in its release from the 7SK snRNP [16]. Similarly, activation of the PI3K/Akt pathway through treatment of cells with HMBA results in the phosphorylation of the cyclin T1 binding region of HEXIM1 and leads to the global release of P-TEFb *in vivo* [17]. Finally, an analysis of active P-TEFb in the cell showed that free low molecular weight P-TEFb is acetylated, while P-TEFb bound to the 7SK snRNP is not, suggesting that acetylation could cause release of P-TEFb from the 7SK snRNP [18]. In addition to post-translational modifications, there is evidence that cellular proteins recruit P-TEFb to sites of active transcription. These include the p65/RELA subunit of NF-κB [19], CIITA [20], [21], Myc [22], [23], MyoD [24], [25], the androgen receptor [26], [27], the estrogen receptor [28], and the bromodomain containing protein Brd4 [29], [30]. However, it is not known if any of these enzymatic modifications or protein interactions liberates P-TEFb directly from the 7SK snRNP.

At least four viral proteins have been shown to recruit P-TEFb to their promoters through an interaction with cyclin T1. These include EBV E2, HSV VP16, HTLV Tax and HIV-1 Tat [31], [32], [33], [34]. Early work on the functional domains of Tat showed that a cysteine rich region is required for Tat transactivation [35], [36]. It was originally thought that this region was important for Tat dimerization, but it is now known that it is the P-TEFb binding domain [34], [37], [38]. The ability of Tat to bind to the TAR element through its basic RNA binding domain is also important for viral replication, because loss of this region results in a significant reduction in HIV Tat transactivation [36], [39]. Also of interest, the bulge sequence that Tat binds to in TAR (AUCUG) is repeated 3 times in the first 100 bases of 7SK RNA and seems unlikely to be a mere coincidence considering the fact that Tat has a greater affinity for 7SK than TAR in vitro [40]. It has been shown that Tat can compete for HEXIM1 binding to P-TEFb and that the cysteine rich P-TEFb binding region of Tat is required for this to occur in vitro and in vivo [40], [41]. Although details of the interaction between Tat and P-TEFb have been recently revealed by structural studies [38], it is not known if Tat can extract P-TEFb directly from the 7SK snRNP, or if this release is mediated by other proteins.

Since HIV Tat is capable of recruiting P-TEFb to a specific genomic location and viral proteins typically mimic the function of cellular proteins, it seems likely that endogenous proteins perform a similar function in recruiting P-TEFb to specific sites of transcription. One potential candidate is bromodomain-containing protein 4 (Brd4). Brd4 has been shown to interact with many different chromatin and transcription related proteins by binding to acetylated lysines, and most importantly its C-terminal helical region binds directly to cyclin T1 of P-TEFb [42], [43]. In fact, most of the P-TEFb that is not in the 7SK snRNP is found complexed with Brd4 when the proteins are extracted from nuclei at high salt and then the salt is removed [29]. Recent evidence also shows that Brd4, P-TEFb, and HEXIM1 localize to nuclear speckles and this may represent a region of P-TEFb activity or a switch between inactive and active P-TEFb [44]. There is also evidence that Brd4 and P-TEFb appear simultaneously at sites of activation [30], [45]; however, the direct role that Brd4 plays in this process is not fully understood. Because Brd4 associates with a large fraction of P-TEFb and also binds to active chromatin, it is possible that it is part of a general mechanism for functional recruitment of P-TEFb.

The 7SK snRNA plays a critical role in the regulation of P-TEFb. Its discovery as a major structural component of a P-TEFb inhibitory complex was novel [46], [47]. Further characterization of the regions of 7SK required for P-TEFb inhibition were performed and it was found that the 1–100 region of 7SK is bound specifically by HEXIM1 and that the 3′ stem loop is bound to cyclin T1 [13], [48]. Additionally, HEXIM1 photo-crosslinks specifically to U30 of 7SK further underscoring the importance of the 1–100 region in P-TEFb inhibition [49]. The protein composition of the 7SK snRNP is dynamic; however, the La related protein LARP7 is constitutively associated with 7SK [50], [51], [52], [53]. 7SK was found to form multiple RNPs with hnRNPA1, A2/B1, R and Q along with RNA Helicase A [52], [54]. The authors of these studies speculated that hnRNP proteins are important for releasing P-TEFb from the 7SK snRNP by competing with HEXIM1 for 7SK binding. This might be mediated by hnRNP binding to a hairpin loop in the middle of 7SK, which when deleted prevents the release of cyclin T1 from exogenously expressed RNAs [52]. HEXIM1 has been shown to be a promiscuous double stranded RNA binding protein and binds to 7SK in vitro even in the absence of P-TEFb [55], so it is odd that HEXIM1 leaves the 7SK snRNP when P-TEFb is released in vivo. It is possible that HEXIM is displaced by the binding of hnRNP proteins that occurs upon P-TEFb release [51], [52], [54].

## Results

### Release of P-TEFb from the 7SK snRNP by HIV Tat in vitro

**Figure 1:**
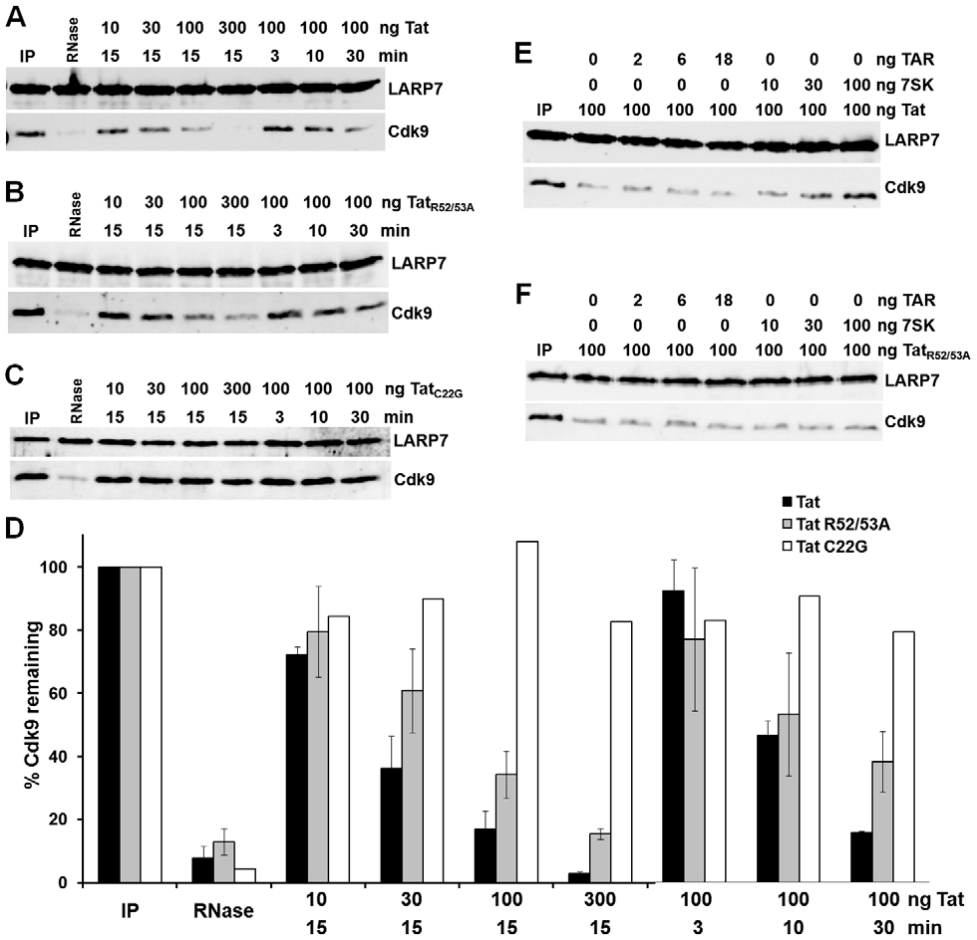
Release of P-TEFb from the 7SK snRNP by HIV Tat. A) P-TEFb release reactions were performed as described in Materials and Methods. Indicated amounts of Tat were incubated for the indicated times with immunoprecipitated 7SK snRNPs. B) Same as in A except the RNA binding deficient mutant of Tat (Tat R52/53A) was used. C) Same as in A except the P-TEFb binding deficient mutant of Tat (Tat C22G) was used. D) Tat release was quantified from three independent experiments for wild type Tat and the RNA binding mutant and two independent experiments for the P-TEFb binding mutant. Mean values for the percent of Cdk9 left in the complex are plotted and all error bars represent standard error. E) 100 ng of Tat was incubated with the indicated amounts of TAR or 7SK RNA before being added to the 7SK snRNP. F) Same as in E except TatR52/53A was used.

Overexpression of Tat in vivo results in the loss of P-TEFb from the 7SK snRNP in the absence of any other HIV proteins, RNA or DNA and in vitro Tat can bind to 7SK RNA and compete with HEXIM1 for binding [40]. However, the ability of Tat to directly extract P-TEFb from the 7SK snRNP has not been explored. To test this, Tat was expressed and purified from *E. coli*. The 7SK snRNP was then isolated from HeLa cell lysates using affinity purified antibodies to LARP7 covalently attached to paramagnetic beads. The isolated complexes were washed, aliquoted, and recombinant protein was then incubated with the 7SK snRNP for 15 minutes before the beads were concentrated and analyzed by Western blot for LARP7 as a control for loading and for Cdk9 to detect release of P-TEFb. As a positive control, RNase A was added to one reaction. Degradation of 7SK destroys the link between LARP7 and P-TEFb and as expected RNase caused essentially all Cdk9 to be released from the complex (Figure 1A). Addition of 10, 30, 100, or 300 ng of Tat for 15 minutes resulted in the graded release of P-TEFb from the 7SK snRNP (Figure 1A). The kinetics of Tat release was also analyzed by exposure of the 7SK snRNP to 100 ng of Tat for 3, 10, or 30 minutes. More P-TEFb was released as exposure time increased. Evidently, Tat can cause the release of P-TEFb directly from the 7SK snRNP in the absence of soluble cellular factors.

The effect of the mutation of the P-TEFb binding or RNA binding domains of Tat on P-TEFb release was also determined. The first 48 amino acids of Tat forms extensive contacts with P-TEFb by directly interacting with mainly cyclin T1 and with two Zn atoms coordinated by the complex [34], [38]. The remainder of the 86 amino acid protein is basic and interacts with TAR RNA [56]. Both domains are required for recruitment of P-TEFb to TAR and for Tat transactivation in vivo. Of interest, the bulge sequence that Tat binds to in TAR (AUCUG) is repeated 3 times in the first 100 bases of 7SK RNA. It was hypothesized that, in addition to the ability of Tat to bind to P-TEFb, a competition with HEXIM1 for binding to 7SK may also be important for Tat mediated P-TEFb release [40]. To determine the effect of the loss of the P-TEFb binding region, cysteine 22 that is critical for Zn coordination was mutated to glycine (Tat C22G). An RNA binding mutant was generated by changing two arginines that are essential for RNA binding region to alanines (Tat R52/53A).

The effect of the loss of the RNA binding activity of Tat on P-TEFb release was tested by titrating Tat R52/53A into reactions containing the 7SK snRNP. Although addition of increasing amounts of the RNA binding mutant resulted in a graded loss of Cdk9 from the 7SK snRNP, the relative amount of release was less than that caused by wild type Tat (Figure 1B). The same kinetic experiment as before was performed and a similar, although slower loss of Cdk9 was observed (Figure 1B). The role of the P-TEFb binding domain of Tat in P-TEFb release was then explored. Addition of increasing amounts of Tat C22G or incubation of the 7SK snRNP with 100 ng of Tat C22G for 30 minutes abrogated the P-TEFb release activity seen with the wildtype protein indicating that the P-TEFb binding region is required (Figure 1C). Furthermore, since the RNA binding region of Tat was still intact in this mutant, this experiment also shows that the RNA binding region is not sufficient to release P-TEFb from the 7SK snRNP. The P-TEFb release experiments were repeated a number of times and the results quantitated. The average percent of P-TEFb remaining in the complex after treatment with wildtype and mutant Tats is plotted in Figure 1D. It is clear that the P-TEFb binding domain of Tat is required for release of P-TEFb from the 7SK snRNP and that the RNA binding domain plays a lesser, but not insignificant role in the release.

Because the preceding results suggested that the RNA binding region may play role in P-TEFb release from the 7SK snRNP and because Tat binds preferentially to 7SK RNA over TAR RNA in an in vitro assay [40], we tested the effect of adding RNAs to the release assay. This was done by pre-incubating 100 ng of Tat with increasing amounts of HIV TAR or 7SK RNA for 15 minutes before the 7SK snRNP was added to these reactions. Interestingly, TAR RNA had no effect on the ability of Tat to release P-TEFb; however, 7SK RNA inhibited it, again indicating that Tat binding to 7SK RNA may be important for its ability to release P-TEFb and confirming that it has a greater affinity for 7SK than TAR (Figure 1E). To determine the effect of the loss of the RNA binding domain on P-TEFb release, the same experiment was performed except this time Tat R52/53A was used. In contrast to wildtype Tat, release of P-TEFb by Tat R52/53A was not inhibited by the presence of 7SK RNA (Figure 1F). These results suggest that Tat binding to 7SK snRNA may play role in P-TEFb release from the 7SK snRNP, but the RNA binding region is neither essential nor alone sufficient to cause release.

### Release of P-TEFb from the 7SK snRNP by the C-terminal region of Brd4

**Figure 2:**
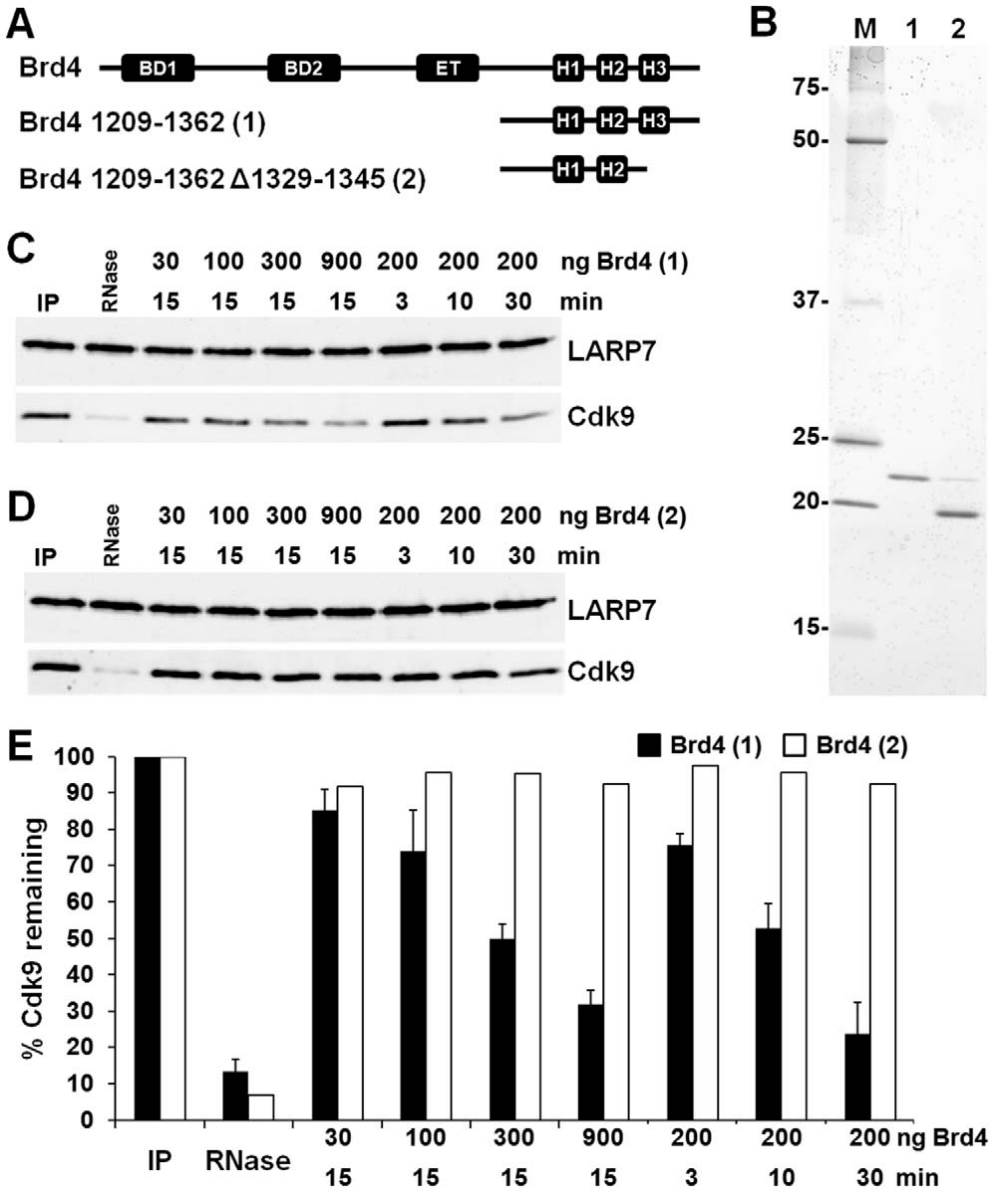
Release of P-TEFb from the 7SK snRNP by the P-TEFb binding domain of Brd4. A) A schematic of Brd4 constructs used. BD1, Bromodomain 1; BD2, Bromodomain 2; ET, Extraterminal domain; H1, H2 and H3, Helical domains 1, 2 and 3. Brd4 1209–1362 contains three helical regions, Brd4 1209–1362 Δ1329–1345 is missing helical region 3 which is required for P-TEFb binding. B) Recombinant protein expressed and purified from *E. coli*. A contaminating band can be seen in the Brd4 mutant preparation and this was confirmed to be an *E. coli* protein by Western blot (data not shown), not full length Brd4. M, Marker; 1, Brd4 1209–1362; 2, Brd4 1209–1362 Δ1329–1345. C) Indicated amounts of Brd4 were added into the release reaction for the indicated times. D) Same as in C except the Brd4 mutant missing helical domain 3 was used for the reactions. E) Brd4 release was quantified from three independent experiments. Two independent experiments were done to calculate the mean for the Brd4 helical domain 3 mutant. The y-axis is a measure of percent of Cdk9 left in the complex. All error bars represent standard error.

Because the viral protein Tat was able to release P-TEFb directly from the 7SK snRNP, we hypothesized that an endogenous cellular protein might possess a similar activity. Brd4 was screened as a potential P-TEFb release factor because under some extraction conditions Brd4 is associated with the majority of non-7SK bound P-TEFb in the cell and it has a known P-TEFb binding domain [29], [30], [43]. The P-TEFb binding region was originally identified to be in an N-terminal region containing one of the two bromo domains [29], but this was later attributed to a cloning error [43]. The P-TEFb binding region is actually encoded by helical region 3 in the extreme C-terminal region of Brd4 [43]. Additionally, two labs have shown that over expression of the C-terminal tail of Brd4 can inhibit Tat transactivation of the HIV LTR [42], [43] and block Tat binding to P-TEFb [43]. cDNAs encoding the P-TEFb binding region (amino acids 1209–1362) and a P-TEFb binding mutant lacking helical region 3 (1209–1362 Δ1329–1345) of Brd4 were obtained from the Verdin Lab (Figure 2A). Both proteins were cloned into pET21a with a C-terminal histidine tag and then expressed and purified from *E. coli* (Figure 2B). Addition of increasing amounts of Brd4 1209–1362 resulted in the obvious release of Cdk9 from the 7SK snRNP (Figure 2C). Kinetic release of Cdk9 with 200 ng of Brd4 was also observed implicating Brd4 as a cellular P-TEFb release factor. Finally, the Brd4 mutant lacking the C-terminal helical domain 3 of Brd4 was used in the release assay and had no effect on Cdk9 release either by titration or time course (Figure 2D). The Brd4 release data quantified from three independent experiments shows that the only the intact P-TEFb binding domain of Brd4 causes a significant release of P-TEFb from the 7SK snRNP (Figure 2E).

**Figure 3:**
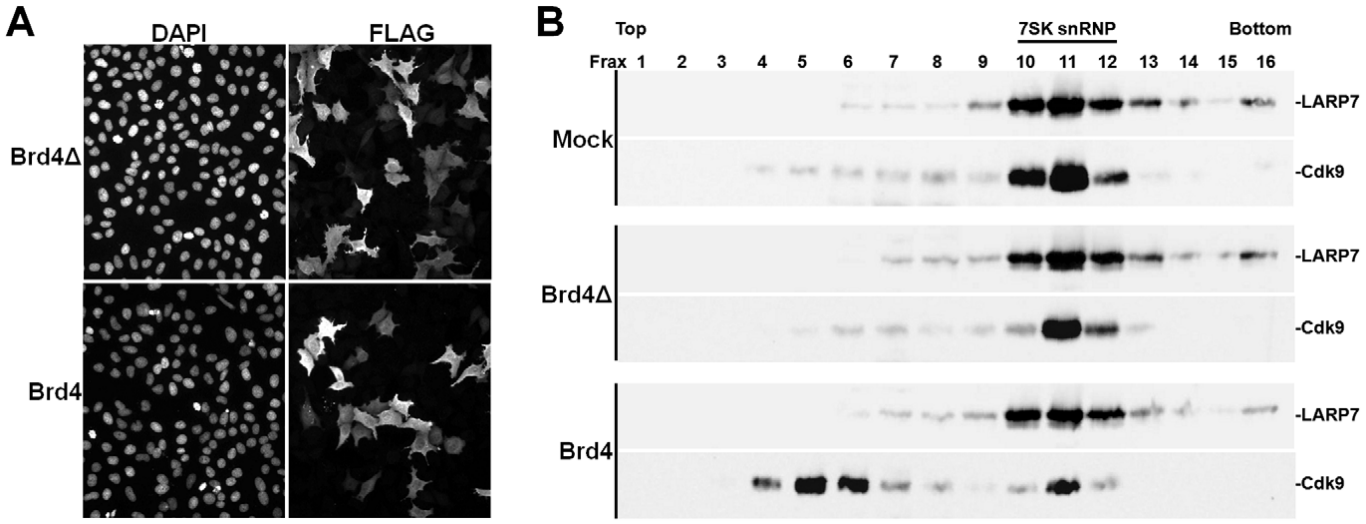
The P-TEFb binding region of Brd4 causes a release of P-TEFb from the 7SK snRNP in vivo. HeLa cells were transfected with plasmids expressing Brd4 1209–1362 (Brd4) or mutant Brd4 1209–1362 Δ1329–1345 (Brd4Δ). A) Immunofluorescence microscopy. 48 hours after transfection cells were fixed and stained for DNA (DAPI) or the FLAG tagged Brd4 constructs (FLAG). B) Glycerol gradient sedimentation analysis. Cell lysates were prepared 48 hr after transfection and sedimented on 5–45% glycerol gradients as described in Materials and Methods. Western blots of fractions were probed with antibodies to LARP7 or Cdk9 as indicated.

To determine if the P-TEFb binding domain of Brd4 would cause the release of P-TEFb from the 7SK snRNP in vivo, HeLa cells were transfected with plasmids that led to the expression FLAG tagged versions of either the wildtype P-TEFb binding domain of Brd4 (1209–1362) or the mutant domain (1209–1362 Δ1329–1345). Transfection efficiencies were similar for the two proteins and greater than 50% as evidenced by immunofluorescence microscopy using antibodies against the FLAG epitope (Figure 3A). High levels of both proteins were found in the nucleus and cytoplasm (Figure 3A). Whole cell lysates were generated from mock transfected cells as well as the two Brd4 transfected cells and analyzed by glycerol gradient sedimentation. As expected, Western blot analysis of LARP7 and Cdk9 from the control cell lysate indicated that most of the P-TEFb was in the more rapidly sedimenting 7SK snRNP (Figure 3B, frax 10–12). An identical pattern was obtained from the Brd4Δ expressing cells (Figure 3B). However, expression of Brd4 (1209–1362) had a dramatic effect in that most of the P-TEFb was now found in slower sedimenting fractions (Figure 3B frax 4–6). As has been found before [51], LARP7 sedimentation was unaffected by release of P-TEFb. These *in vivo* findings support the *in vitro* results and strongly implicate Brd4 in the release of P-TEFb from the 7SK snRNP.

### Release of P-TEFb is accompanied by a large conformational change in 7SK RNA

**Figure 4:**
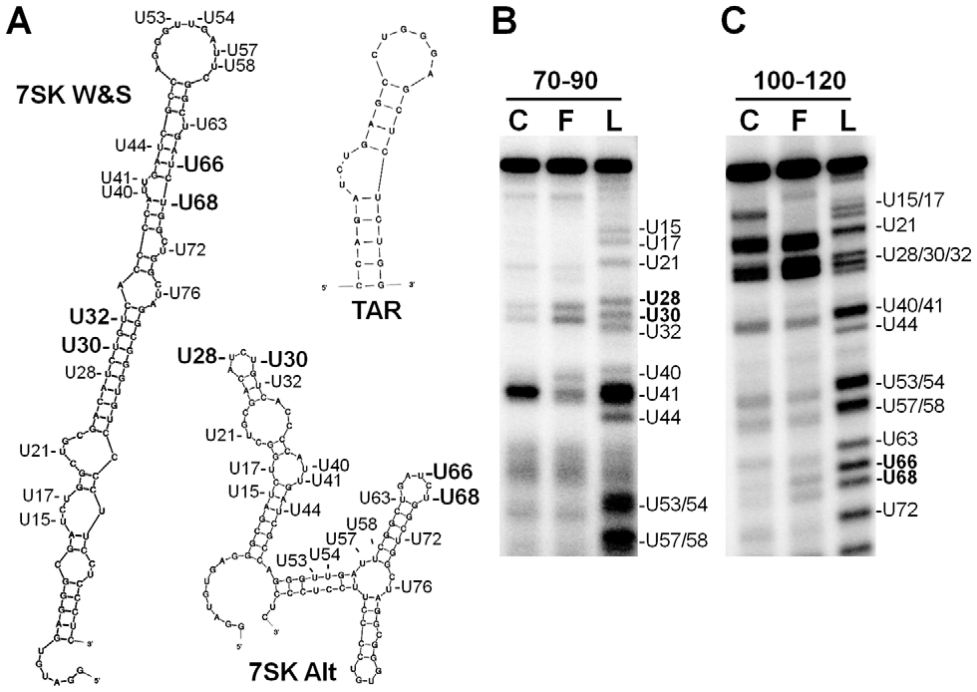
Release of P-TEFb by flavopiridol treatment of cells causes a conformational change in 7SK snRNA. A) HIV-1 TAR RNA stem loop: 35–61 of TAR RNA covering the Tat binding bulge (AUCUG) and the cyclin T1 binding loop (CUGGG). 7SK Alt: Predicted structure of the 1–100 region of 7SK. 7SK W&S: structure of the 1–100 region of 7SK RNA described previously [57]. The uracil residues in 7SK RNA are marked. B) and C) CMCT reactivity was determined as described in the Materials and Methods using extension of the indicated primers. C, control cells; F, flavopiridol treated cells; L; sequencing ladder showing the position of uracils.

Re-analysis of the original data used to characterize the structure of 7SK revealed that 7SK may exist in more than one conformation in the cell. Based on chemical modification and nuclease sensitivity studies, Wassarman and Steitz created a best fit structure for 7SK [57]. They placed uracils 28 and 30, and uracils 66 and 68, which are in the important HEXIM binding 1–100 region of 7SK, in double stranded regions even though these bases showed sensitivity to the uracil modifying agent CMCT (Figure 4A, 7SK W&S). CMCT should be able to bind to and modify any bases located in accessible loops because CMCT is a small molecule that reacts specifically with N-3 of uracil. Since these bases were moderately sensitive, we hypothesized that 7SK snRNA exists in more than one conformation in the cell. Although the 7SK snRNP inhibits the majority of P-TEFb, a significant fraction of this complex is not normally associated with P-TEFb [51]. This suggests that there are at least two 7SK snRNPs, one with and the other without P-TEFb, which may stabilize different structures of 7SK.

To determine if different structures were computationally possible, the first 100 bases of 7SK snRNA were folded using the RNA/DNA folding program mFold v2.3 [58]. A number of structures were generated, but many of the structures with low energy fell into two ensembles of similar structures. Both groups were represented in the top two most stable structures. One was identical to the structure described by Wassarman and Steitz (Figure 4A, 7SK W&S, ΔG −40.1 kcal/mole) while the second folded into an alternate, slightly more stable structure (Figure 4A, 7SK Alt, ΔG −40.4 kcal/mole). The specific alternative structure shown is used for illustrative purposes and we do not mean to imply that the exact structure shown in Figure 4 is present in 7SK snRNP. Interestingly, in the alternate structures U28, U30, U66, and U68 are found in CMCT accessible loops. A mixture of the two forms would more adequately explain the Wassarman and Steitz data.

To test if this second alternative structure of 7SK RNA was physiologically relevant, the structure of 7SK RNA before and after P-TEFb release by flavopiridol was probed by primer extension of CMCT treated 7SK snRNPs. This was done by scaling up the immunoprecipitation protocol used for the release assays to obtain 400 ng of RNA for 7SK structure analysis. Two liters of HeLa cells were grown in spinner flasks, split in half and treated with either DMSO carrier as a control or with flavopiridol to release P-TEFb from the 7SK snRNP. Lysates were made, LARP7 immunoprecipitation was performed, isolated complexes were washed, and then the complexes were treated with CMCT to record the structure of 7SK locked in the 7SK snRNP. The RNA was isolated and then primer extension was performed using AMV reverse transcriptase and a radiolabeled DNA primer to the 100–120 region or to the 70–90 region. Modification of uracil by CMCT blocks reverse transcription beyond the modification site and results in the production of an RNA sensitivity ladder. The 70–90 primer was used to obtain more band separation in the 1–50 region to better visualize CMCT sensitivity at U28 and U30. Release of P-TEFb from the 7SK snRNP by flavopiridol treatment of cells resulted in significant changes in the structure of 7SK. U28, U30 (Figure 4B), U66, and U68 (Figure 4C) all became more sensitive to CMCT after P-TEFb release compared with the untreated control. These results confirm that the alternative structure is similar to the structure of the P-TEFb-free form of 7SK RNA (Figure 4A). Additionally, in the alternative structure, U40/U41, U53/U54, and U76 remain in single stranded regions and their sensitivities do not change significantly after P-TEFb release, further supporting the existence of a conformational switch in 7SK after P-TEFb release (Figure 4C).

**Figure 5:**
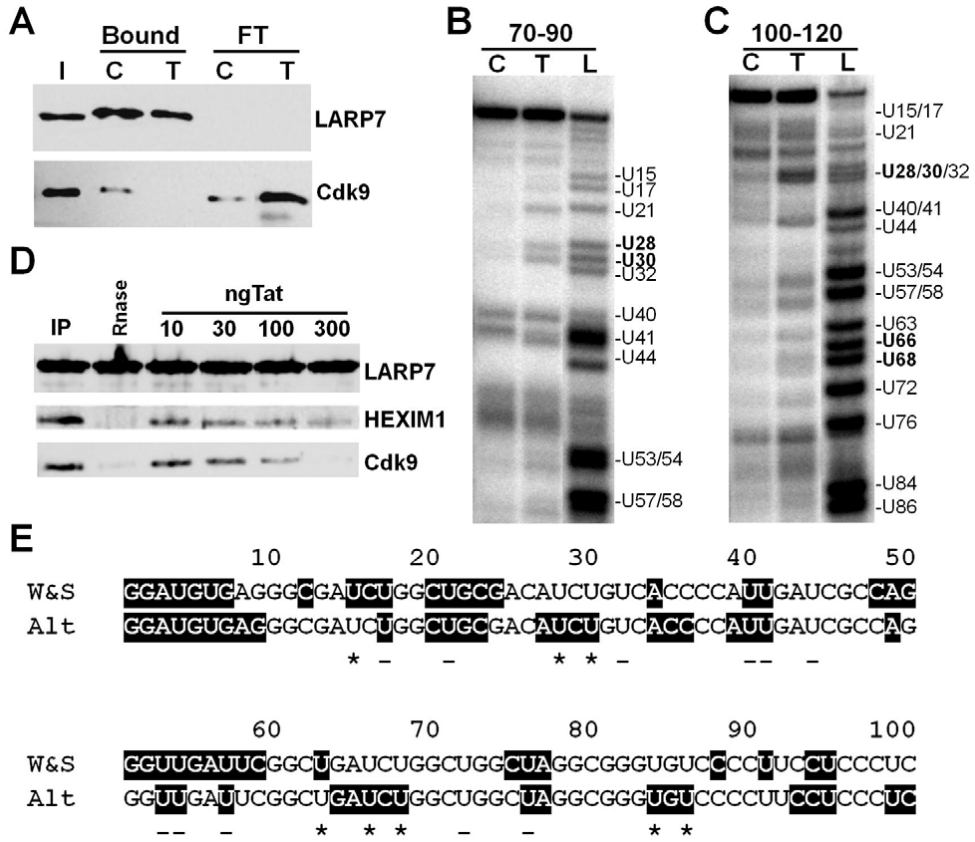
P-TEFb release by HIV Tat causes a conformational change in 7SK snRNA and release of HEXIM1 from the complex. A) Western analysis of P-TEFb release from the 7SK snRNP after co-incubation of Tat. LARP7 and Cdk9 were analyzed by Western. I, Input; C, Control; T, Tat treated; Bound, bound to the beads; FT, flowthrough from the beads. B) and C) CMCT reactivity was determined as described in the Materials and Methods using extension of the indicated primers. D) Western blot of the Tat treated 7SK snRNP probed for Cdk9 and HEXIM1. Indicated amounts of Tat were added to the 7SK snRNP. E) Graphical summary of the 7SK conformational change data. Bases in single stranded regions are boxed in black and bases in double stranded regions are unboxed. An asterisk denotes a change in CMCT reactivity and a minus sign indicates no change.

To determine if this conformational change occurred after P-TEFb release by other agents, lysates were again made except one was incubated with 10 µg/mL of Tat during the LARP7 immunoprecipitation. Tat was used because it can extract P-TEFb directly from the 7SK snRNP and because U28, U30, U66, and U68 all reside in AUCUG repeats on 7SK (Figure 4A). Incubation of the lysate with Tat during the immunoprecipitation caused the release of P-TEFb from the 7SK snRNP (Figure 5A). As was observed with flavopiridol release, U28, U30, U66, and U68 all became more sensitive after P-TEFb left the complex (Figure 5B and 5C). To determine if Tat also caused HEXIM1 to be released at the same time as P-TEFb in vitro, the LARP7 immunoprecipitates shown in Figure 1A were also probed for HEXIM1. As was found for Cdk9, decreasing amounts of HEXIM1 were found as the amount of Tat was increased (Figure 5D). However, release of HEXIM1 did not reach the same extent as Cdk9 suggesting that HEXIM1 release might lag the release of P-TEFb. The results of these CMCT modification experiments support the idea that a conformational change occurs in the structure of 7SK RNA after P-TEFb leaves the 7SK snRNP. The 7SK structural change data at the uracil positions are summarized in Figure 5E. Every U residue that changed in reactivity to CMCT upon loss of P-TEFb from the 7SK snRNP was found in a region that changed its conformation from single to double or from double to single stranded. All U residues that didn't change in reactivity upon loss of P-TEFb were found in regions that also did not change.

## Discussion

Because most human genes contain promoter proximally paused polymerases [10], [11], the goal of this study was to understand how the factor responsible for release of these poised polymerases into productive elongation, P-TEFb, is targeted to specific genes. Most of the P-TEFb in the cell is bound to and inhibited by HEXIM1 found in the 7SK snRNP [59]. We demonstrated that two activators of transcription, HIV-1 Tat and human Brd4, could extract P-TEFb from the 7SK snRNP in vitro in the absence of other soluble cellular factors or enzymes. As was found previously for Tat [40], the P-TEFb binding domain of Brd4 was also able to cause the release of P-TEFb from the 7SK snRNP in vivo. Finally, we were able to resolve the mystery as to why HEXIM1 was ejected from the 7SK snRNP after release of P-TEFb. These results provide strong evidence for the idea that activators take the P-TEFb they need from the 7SK snRNP and that the process is highly regulated.

### Model for activator mediated release of P-TEFb from the 7SK snRNP

**Figure 6:**
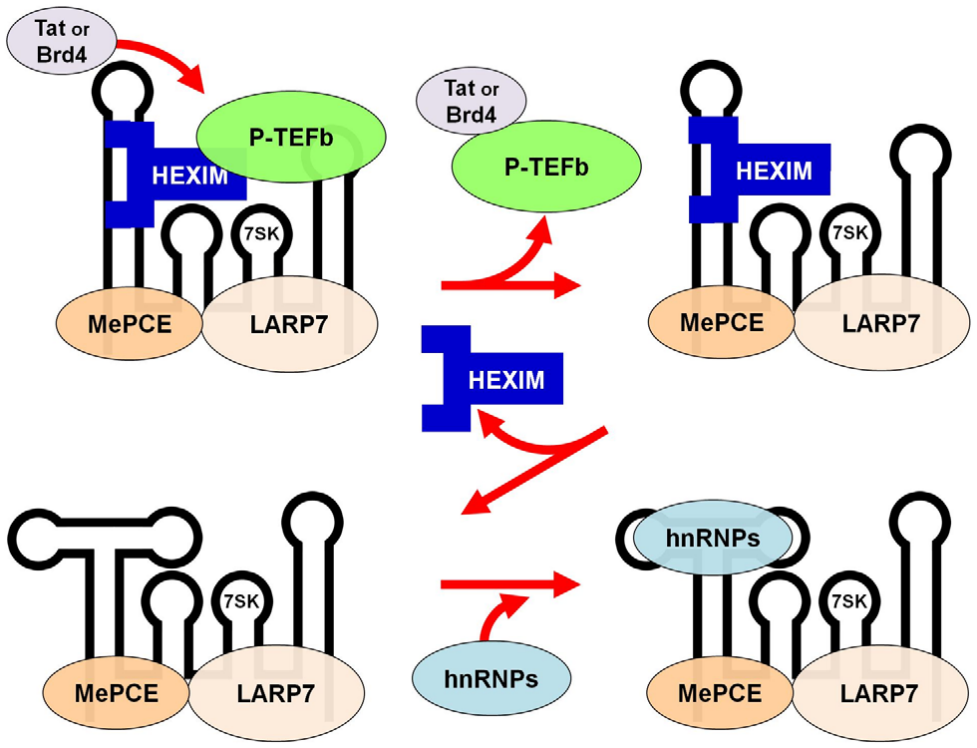
Model of P-TEFb and HEXIM1 release from the 7SK snRNP. P-TEFb is directly extracted from the 7SK snRNP by Tat or Brd4. This leads to the loss of P-TEFb, a destabilization of the 7SK structure resulting in a conformational change in the RNA that causes HEXIM1 to be released from the 7SK snRNP. hnRNP proteins then bind to the region of RNA unmasked by the loss of HEXIM1.

Based on our results here and on published studies, we propose a more refined model for release of P-TEFb from the 7SK snRNP (Figure 6). Tat, Brd4 or another P-TEFb extractor binds directly to P-TEFb and removes it from the complex. This leaves HEXIM1 bound to the 7SK snRNP. In the second step, HEXIM1 is released from the RNA and 7SK rearranges into an alternative conformation. Loss of HEXIM1 could be caused by the rearrangement of 7SK or the rearrangement could occur after HEXIM1 dissociates. What is clear is that the equilibrium between free and RNA bound HEXIM1 lies strongly in favor of free HEXIM1 after P-TEFb is lost from the complex. P-TEFb has affinity for 7SK and HIV TAR RNA [37], [48] and it is possible that this helps stabilize the binding of HEXIM1 to 7SK in the presence of P-TEFb. After P-TEFb and HEXIM1 are released the 7SK snRNP associates with a number of hnRNP proteins [51], [52], [54], [60]. Presumably this further stabilizes the P-TEFb/HEXIM-free 7SK snRNP.

While not explicitly shown in the model, this entire process must be reversible and is likely also highly controlled. An RNA helicase was found in a proteomic analysis of the 7SK snRNP [51], [52], [61]. It does not seem to be involved in the release of P-TEFb in vitro because ATP is not required. However, it could be involved in the re-assembly process. The re-association of HEXIM1 with the 7SK snRNP must be regulated because in HeLa cells 80% of HEXIM1 is free from 7SK and about half of the 7SK in the cells lacks HEXIM1 and P-TEFb [13], [14], [51]. Because our in vitro release assay does not contain excess hnRNPs and HEXIM1 dissociates after P-TEFb is released, simply removing the hnRNPs would not be enough to cause HEXIM1 re-association. P-TEFb is unable to bind to HEXIM1 unless the dsRNA binding domain of HEXIM is occupied [55]. Binding to RNA leads to a conformational change in HEXIM1 that allows P-TEFb to bind [55]. Therefore, the most likely step to regulate in the re-assembly of the inactive P-TEFb/HEXIM1 7SK snRNP is the binding of HEXIM1. This could be controlled by regulating the conformation of 7SK or by post translational modification of HEXIM1. Phosphorylation of HEXIM1 has been demonstrated to block binding to RNA [17], but it is not clear if this is the major mechanism for controlling reassembly of the snRNP.

A key feature of our model is the conformational rearrangement of 7SK. In the absence of a crystal structure, the exact conformation(s) of 7SK in snRNPs can only be approximated by the available methods. For our study we chose chemical modification by CMCT because it offered a direct test of the environment around U30 that is known to be in the close proximity to bound HEXIM1 [49]. Use of the small chemical modification reagent also circumvented the issues of limited accessibility to protein bound regions when large nucleases are used to probe RNA structure. We found clear evidence of a change in the HEXIM1 binding region from double to single stranded character when P-TEFb was released from the 7SK snRNP. Although the proposed alternative structure (see Fig. 4) fits our data, as described earlier, we are not proposing that the exact alternative structure shown is that which is found in vivo. In a detailed examination of 7SK RNA throughout evolution Marz et al. proposed an alternative structure for 7SK [62]. That structure differs from both the Wassarman and Steitz structure and our alternative structure in that it contains a short stem (M1) that forms between the 5′ end of 7SK and a region just upstream from the final stem loop about 300 nucleotides downstream. The M1 stem is picked up by mFold, but only when the entire 7SK sequence is input. However, the Marz structure more closely matches the Wassarman and Steitz structure in the HEXIM1 binding region except that U30 (but not U28, U66 and U68) is opposite a very short stem loop and is unpaired in the Marz structure.

The 7SK conservation study identified three regions that could form alternative pairings with the M2 stem [62], therefore, a rearrangement in this region could act as a trigger by disrupting the structure at the base of the large stem loop to which HEXIM1 binds leading to a conformational change of the HEXIM1 binding region. In support of this possibility the reactivity of U21 which is in this region was seen to change slightly during Tat mediated P-TEFb release (see Fig. 5). LARP7 would be a prime candidate for regulating this process. By analogy to the properties of La, the La domain in the N-terminus of the protein is almost certainly involved in association of LARP7 with the 3′ end of 7SK [63]. The hypothesized M1 loop [62] would bring the 3′ and 5′ ends of 7SK into close proximity of each other and another RNA binding domain present in the C-terminal region of LARP7 could be involved in stabilizing or destabilizing the M1 loop or one particular arrangement of the M2 stem.

### Role of the RNA binding domain of Tat

When HIV infects a cell it not only takes over P-TEFb through association with Tat, but also takes over the control of P-TEFb by the 7SK snRNP. The RNA binding domain of Tat is required for efficient transactivation in vivo [36] and was originally thought to be utilized just in recruiting P-TEFb to TAR element in the nascent HIV transcript. Early models had Tat binding to TAR and then recruiting P-TEFb [34], [64], [65], [66]. Now we know that Tat can take P-TEFb directly from the 7SK snRNP in the absence of TAR or other viral proteins and that the Tat•P-TEFb complex is what is recruited to TAR [40]. Several pieces of evidence point toward a role of the RNA binding domain of Tat in the release of P-TEFb from the 7SK snRNP. First, it binds better to 7SK RNA than to TAR, and in a defined in vitro system with just 7SK and HEXIM1, Tat can displace HEXIM1 [40]. This may involve one or more of the three AUCUG sequences found in 7SK that are identical to the sequence in TAR that is essential for Tat binding to TAR. An AUCUG sequence is found in a conserved location in almost all putative 7SK sequences found across all species with a 7SK homologue suggesting that it is involved in a critical function of 7SK [62]. Second, we showed here that a Tat mutation that abrogated RNA binding slowed P-TEFb release and, importantly, addition of excess of 7SK RNA to release reactions with wildtype Tat blocked the ability of Tat to release P-TEFb. The RNA binding domain may play a non-essential, but potentially significant role in release of P-TEFb from the 7SK snRNP. The P-TEFb binding region of Brd4 has not been shown to bind to RNA, so perhaps cellular activators do not require RNA binding.

We speculate that HIV may have evolved to take over the control of P-TEFb by events that occurred during the integration of an early form of the virus. The human genome has thousands of copies of 7SK pseudogenes. Perhaps a viral genome became juxtaposed with a 7SK sequence during a particular integration event. The virus might have found it advantageous for persistence to have the HEXIM1-mediated repressive function of a 7SK sequence keeping the virus in an inactive state. The virus then could have created Tat by modifying a HEXIM protein so that it eliminated the P-TEFb inhibitory domain. According to this idea, the HIV 5′ UTR maintained HEXIM binding properties to help maintain latency, but with Tat had a way to activate the LTR to extremely high levels under appropriate cellular conditions. During this evolutionary process Tat had to deal with not only the 7SK like sequence in the LTR, but also with the cellular 7SK snRNP. The end product is a very finely tuned system using cellular mechanisms controlling P-TEFb to the advantage of the virus. A second driving force likely used during evolution was the effect that Tat had on blocking other activators ability to recruit P-TEFb. Tat competes with the co-activator CIITA for binding to P-TEFb and this leads to down regulation of MHC class II genes during infection [20]; a clear advantage for the virus. Also Tat and Brd4 have been shown to compete for P-TEFb and this could have global effects on gene expression [42], [43]. Attesting to the uniqueness of this evolution, HIV and other highly similar viruses utilize the only known activator (Tat) that is targeted to a specific gene through RNA.

### Significance for regulation of gene expression

A large body of evidence points to the P-TEFb dependent step in transcription as one of the most highly utilized in regulating gene expression. About 80% of human genes and a large fraction of Drosophila genes have RNA polymerase II molecules poised in promoter proximal positions ready for P-TEFb to release them into productive elongation that ultimately leads to the generation of mature mRNAs [6], [10], [11]. Also the expression of essentially all genes has been shown to require P-TEFb [11], [67]. It is no wonder that most P-TEFb is carefully tied up in the 7SK snRNP. Globally releasing it could catastrophically affect the pattern of genes expressed by inappropriately activating most genes. The biochemical properties of the 7SK snRNP are highly suited for a function in which P-TEFb is released only near genes that should be expressed. First, unlike all other snRNPs the 7SK snRNP is completely extracted by low salt conditions from mild detergent treated nuclei [14]. This demonstrates that it is not tightly associated with any nuclear structures and is, therefore, free to diffuse around the nucleus. This property allows the potentially dangerous kinase activity of the factor to be nearby any gene, but held in check by HEXIM1. All that is needed is a mechanism to extract the P-TEFb and keep it on task at specific genes, and we have demonstrated here that such a mechanism exists. Although Tat directs extracted P-TEFb to the HIV LTR, Brd4 extraction of P-TEFb from the 7SK snRNP provides a general mechanism for recruitment of P-TEFb to active chromatin. Besides Brd4, many other activators and more recently the ELL elongation complex [68] have been shown to associate with P-TEFb on active genes. Myc, a known transcription activator, was not able to release P-TEFb in this assay (data not shown); however, it may function with other protein complexes. A similar interaction has been described for the recruitment of P-TEFb through the association of p65 with Brd4 [69]. It remains to be determined how many cellular factors can extract P-TEFb from the 7SK snRNP, but it is clear that once it is extracted it has a number of interactions that could keep it localized to the appropriate genes. Although recent data has suggested that post-translational modification of the components of the 7SK snRNP, such as P-TEFb acetylation [18], HEXIM phosphorylation [17], or P-TEFb dephosphorylation [16] cause the release of P-TEFb from the 7SK snRNP, these modifications had no effect on P-TEFb release in our in vitro assay (data not shown). These modifications could have significance in the context of the nuclear milieu.

Our understanding of how P-TEFb activity is directed to specific genes will benefit from a number of possible avenues of future research. One concerns the re-sequestration of P-TEFb after its job is complete on any particular gene. Perhaps there is a sequential process by which P-TEFb is obligatorily returned to the 7SK snRNP at the end of each transcription cycle. Alternatively, if active genes are looped or clustered then it is possible that P-TEFb is allowed to function on the same gene again or on other active genes before it is inactivated. Support for such regional localization of P-TEFb was recently provided by the Rice lab who found that P-TEFb, HEXIM1 and Brd4 co-localized to nuclear speckles [44]. As discussed above the mechanism of HEXIM1 and P-TEFb re-entry into the 7SK snRNP and the requisite change in the conformation of the RNA deserves further study. As discussed earlier potential regulators of this process include other components of the 7SK snRNP. LARP7 is always associated with at least the 3′ end of 7SK and the methyl phosphate capping enzyme, MePCE, modifies the 5′ end of 7SK. By analogy to the founding member of the La family of related proteins [63], [70], [71], [72], [73], post-translational modifications of LARP7 may result in a reduced affinity the protein for regions of 7SK. Finally, it is known that after P-TEFb and HEXIM leave in vivo that hnRNP proteins along with an RNA helicase bind to the 7SK snRNP [51], [52]. The hnRNP proteins may serve to protect 7SK and stabilize the HEXIM unbound state, while the activity of the helicase may be regulated and activated to convert 7SK back to the HEXIM accessible conformation.

## Materials and Methods

### Expression and Purification of Recombinant Proteins

HIV Tat mutants were cloned into pET21a (Novagene) *E. coli* expression vectors with histidine tags. *E. coli* BL21 star (DE3) cells were transformed with the pET21a clone, used to inoculate 2 liters of LB, gown to an OD_600_ of 0.5 and then expression was induced with IPTG overnight at 18°C. Cells were harvested, washed, and then lysed by sonication. Sonicated lysates were cleared by centrifugation at 200 k × g in a Beckmann Ultracentrifuge for 45 minutes. Nickel resin (Invitrogen) was then added to the cleared lysates and the protein was bound to the resin while rotating at 4°C for 1 hr. The resin was washed and the protein was eluted by addition of imidizole to the nickel resin. The elution was then cleared by centrifugation to remove any precipitated proteins and loaded onto the FPLC. Both mutant Tat proteins are positively charged, so they were purified by FPLC after binding to a Mono S column. The protein was then eluted over a salt gradient and purity was assessed by silver stain.

Purification of the Brd4 proteins was similar, but because both proteins were charge neutral, neither bound to Mono S or Mono Q. Flowthroughs from the Mono S and Mono Q were dialyzed against PBS for 24 hr at 4°C to remove imidizole and detergents from the protein buffers. Purity of the proteins was determined by SDS PAGE followed by silver staining.

### Release Assay

For the release assays, 2 L of HeLa cells (National Cell Culture Center) were cultured in spinner flasks. Cells were washed and then lysed in Lysis Buffer (10 mM HEPES, 150 mM NaCl, 2 mM MgCl_2_, 10 mM KCl, 0.5% NP−40, 0.5 mM EDTA, 1 mM DTT, 0.1% PMSF, and 1 U/mL Roche EDTA free complete protease inhibitor cocktail) for 10 minutes with intermittent vortexing. Lysates were cleared in a Beckmann ultracentrifuge at 200 k × g for 1 hr. After centrifugation, the lipid layer was discarded and the supernatant was saved and frozen in aliquots at −80°C for later use. The M-270 Epoxy Dynabeads used in this assay were prepared the night before. 2×10^8^ (100 µL) beads were washed 2 times in PBS, then rotated for 10 minutes at room temperature in PBS, and finally washed again in PBS. Beads were then resuspended in 50 µg of affinity purified LARP7 antibody and 1 M ammonium sulfate and incubated overnight (16 hr) at 37°C in a PCR tube. After incubation, the beads were washed 4 times in PBS containing 0.5% BSA. After washing, the beads were resuspended in 300 µl of HeLa lysate (one confluent T-150) and incubated at 4°C for 2 hr in a PCR tube. After immunoprecipitation, the beads were washed once in Lysis Buffer, resuspended in 200 µL PBS containing 0.5% BSA, and then 1×10^7^ (10 µL) beads were aliquotted into microfuge tubes. At this point, potential releasing agents were added to the beads. For the release control, 4 µg of RNase A was added to one aliquot of beads. Beads were incubated for 15 minutes, unless otherwise noted, with gentle resuspension every 3 minutes to keep the beads suspended during the release reactions. Reactions were then washed twice with wash buffer (500 µL), and resuspended in 17 µL SDS loading buffer and resolved by 9% SDS-PAGE.

### Western Analysis

After transfer to nitrocellulose, the membranes were cut and incubated in 0.1% Tween 20, 1× PBS and 3% milk overnight at 4°C with the appropriate primary antibody. Membranes were then washed 3 times in 0.1% Tween 20, 1× PBS and incubated with horseradish peroxidase-conjugated secondary antibody (Sigma). The membranes were then treated with Super Signal Dura West Extended Duration Substrate (Pierce) and imaged using a cooled charge-coupled camera (UVP). The antibodies used for Western analysis were: sheep anti-cyclin T1 (Millipore 06-1289), rabbit anti-Cdk9 (sc-8338; Santa Cruz Biotechnology), affinity-purified sheep anti-HEXIM1 (Millipore 06-1291), and affinity-purified sheep anti-LARP7 [51].

### Chemical Modification of 7SK RNA

For chemical modification of the 7SK snRNP, complexes were isolated similarly to the release assay except New England Biolabs paramagnetic protein G beads were used for the isolation. 400 µL of beads were bound to 50 µg of antibody overnight at 4°C, washed, and then split in half and incubated with 800 µL of HeLa lysate. After immunoprecipitation, the beads were washed 4 times with 1 mL of wash buffer. The isolated and washed complexes were then resuspended in 200 µL of N-Cyclohexyl-N-(β-[N-methylmorpholino]ethyl)carbodiimide p-toluenesulfonate (CMCT) modification buffer which consisted of BMK buffer (80 mM potassium borate pH 8.1, 60 mM KCl, 2 mM MgCl_2_, and 3 µg/µl CMCT (Sigma)) and incubated at room temperature with rotation for 15 minutes. The CMCT stock buffer was 42 mg/mL CMCT in BMK buffer. CMCT modification reactions were then stopped by addition of 300 µL stop buffer (0.3 M sodium acetate, 0.2 M PIPES pH 6.5, and 5 mM EDTA) and placed on ice for 3 minutes. The 500 µL of CMCT modified beads were then transferred to 2 mL microfuge tubes and 1 mL of Trizol was added. To that, 200 µL of chloroform was added and then the tubes were vortexed for 20 seconds. The emulsions were incubated for 3 minutes at room temperature and then spun down at 14 k rpm for 20 minutes at 4°C in a microcentrifuge. After the spin, 1.1 mL of the aqueous phase was added to 900 µL of isopropanol, mixed gently, and incubated at room temperature for 10 minutes to precipitate the RNA. The RNA was then spun down at 14 k rpm for 25 minutes, the supernatant was removed with a pulled glass Pasteur pipette and then the RNA was washed with 300 µL of 80% EtOH. Finally, the supernatant was again removed with a pulled glass Pasteur pipette, the pellets were air dried and then resuspended in 20 µL ddH2O. A sample of the RNA was analyzed by 6% TBE urea acrylamide gel electrophoresis and samples were normalized for 7SK RNA content.

### In vitro Transcription of 7SK RNA

The 7SK RNA sequence was cloned into pCR2.1 (Invitrogen). This plasmid was then used as a template in a PCR reaction to generate the 7SK sequence from a T7 promoter containing primer and a reverse primer. 1 µg of PCR product was then used in an in vitro transcription reaction with recombinant T7 RNA polymerase (Stratagene) according to the manufacturer's instructions. Quality of the in vitro transcribed RNA was determined by resolving the RNA on a 6% TBE urea acrylamide gel.

### Hybridization and Primer Extension Reactions

To map the first 100 bases of 7SK and determine the structure of 7SK RNA, HPLC purified antisense DNA primers were ordered from IDT (100–120R: AGGGACGCACATGGAGCGGT, 70–90R: GGGGACACCCGCCTAGCCAG) and radioactively end labeled with γ−^32^P-ATP using T4 PNK. For reverse transcription, primers were hybridized to either 100 ng of in vitro transcribed 7SK RNA to generate the Sanger sequencing ladder, or to 100 ng of the CMCT modified RNA extracted from in vivo isolated RNP complexes. Reactions were hybridized to 200 femtomoles of radiolabeled primer at 80°C for 10 minutes in hybridization buffer (40 mM HEPES pH 7.6, 5 mM boric acid, 100 mM KCl, 20 µg/mL BSA, and 0.5 U RNaseOUT) and then at 42°C for 15 minutes in a thermal cycler. Extension was initiated by the addition of extension buffer (50 mM Tris pH 8.4, 9 mM MgCl_2_, 10 mM DTT, 20 µg/mL BSA, 100 mM KCl, 1 U AMV reverse transcriptase, and 0.5 U RNaseOUT), and dNTP (1 mM final concentration) or sequencing mix (2.5 mM dTTP, dGTP, dCTP, 1.9 mM dATP, and 0.6 mM ddATP final concentration). Primer extension was run for 1 hr in a thermal cycler at a constant 42°C. 4 µg of RNase A was added to the reactions and incubated for 15 minutes at 42°C because the DNA and RNA form a very strong hybrid which complicates gel analysis. Reactions were then suspended in a total volume of 100 µL of H_2_O containing 20 µg of yeast tRNA carrier. 100 µL of phenol:chloroform:isoamyl alcohol (25∶24∶1 v/v) was added and the samples were vortexed for 15 seconds. The samples were spun down at full speed in a microcentrifuge for 5 minutes at room temperature. 100 µL of the aqueous phase was then combined with 4 µL 5 M NaCl (to a final concentration of 200 mM) mixed, and then 200 µL of 100% EtOH was added to initiate nucleic acid precipitation. The DNA/RNA was then precipitated at −20°C for 30 minutes. After incubation, the DNA/RNA was spun down at full speed in a microcentrifuge for 15 minutes at 4°C. The DNA/RNA pellets were washed with 80% EtOH, spun down at full speed for 5 minutes, air dried, and resuspended in 8 µL urea loading buffer. Radiolabled DNA was resolved on a 10% TBE urea acrylamide gel, dried, exposed for 24 hr to a phosphoimager screen, and then imaged with a Fuji-Film FLA 7000 phosphoimager.

### Glycerol gradient analysis

HeLa cells were grown to 90% confluence in three 150 mm diameter, Nunclon Surface petri dishes (Nunc 168381). One coverslip was placed in each flask to check transfection efficiency by immunofluorescence microscopy. Cells were then mock transfected or transfected with a pcDNA3 vector expressing either Brd4 (1209–1362) or Brd4 (1209–1362 Δ1329–1345) using Lipofectamine 2000 (Invitrogen 11668). Cells were scraped in medium 48 hours post-transfection, spun down at 2000 rpm and then lysed for 10 min on ice in 300 mM KCl, 2 mM MgCL_2_, 50 mM HEPES (pH 7.6), 0.1 mM EDTA, 1 mM DTT, 1/1000 PMSF saturated in isopropanol, 1× EDTA-free complete protease inhibitor cocktail (Roche) and 0.5% Triton ×−100. The lysates were clarified by centrifugation for 5 min. at 13 k rpm in a microcentrifuge prior to fractionation on 5 ml, 5–45% glycerol gradients in the same buffer conditions used during lysis, except that Triton ×−100 and the protease inhibitor cocktail were omitted. Gradients were run at 45 k rpm for 16 hr in a Beckman SW-55Ti rotor before being separated into 16 fractions. Gradient fractions were resolved by 9% SDS-PAGE followed by transfer to 0.45 µm nitrocellulose membranes.

### Immunofluorescence microscopy

Cells were fixed in 3.7% formaldehyde in PBS, permeabilized in 0.5% Triton ×−100 in TBS and then blocked in 2% BSA in TBS. Coverslips were stained with 1/200 dilution of anti-Flag antibody produced in rabbit (Sigma-F7425) in TBS for 1 hr at 37°C incubator and incubated with 1/1000 dilution Alexa Fluor 488 goat anti-rabbit IgG (invitrogen-A11034) in TBS for one more hour at 37°C. Slides were imaged using a Leica DMR microscope with appropriate filters.
